# Abnormal expression of miR-330-3p predicts post-prostatectomy urinary incontinence and regulates the function of urethral fibroblasts by targeting MMP2

**DOI:** 10.1186/s41065-025-00475-8

**Published:** 2025-06-18

**Authors:** Xiaoying Feng, Yuanyuan Mi, Mengye Weng

**Affiliations:** 1 Department of Urology Surgery, Affiliated Hospital of Jiangnan University, Wuxi, 214122 Jiangsu China; 2 Department of Orthopedics, Affiliated Hospital of Jiangnan University, No. 1000, Hefeng Road, Wuxi, 214122 Jiangsu China

**Keywords:** miR-330-3p, MMP2, Post-prostatectomy urinary incontinence, Inflammation, ECM remodeling

## Abstract

**Background:**

Post-prostatectomy urinary incontinence (PPUI) is a common complication for patients with prostate cancer after surgery. MicroRNA-330-3p (miR-330-3p) is down-regulated in stress urinary incontinence patients. However, its clinical role and regulatory mechanism in PPUI remain unknown.

**Objective:**

To assess the clinical significance of miR-330-3p in PPUI and to explore the potential mechanisms via matrix metalloproteinase 2 (MMP2) regulation.

**Methods:**

This study enrolled 135 ageing prostate cancer patients (86 without PPUI, 49 with PPUI). Reverse transcription PCR (RT-qPCR) was utilized to measure the levels of miR-330-3p, while Receiver operating characteristic (ROC) analysis was conducted to evaluate the predictive significance of miR-330-3p for PPUI. The proliferative of human urethral fibroblasts (HUFs) was assessed by Cell Counting Kit-8 (CCK-8) assay, while inflammatory cytokines were quantified via enzyme-linked immunosorbent assay (ELISA) kits. Western blot assay was employed to examine the protein levels of extracellular matrix (ECM) remodeling-related markers. The miR-330-3p/MMP2 interaction was validated by dual-luciferase assay.

**Result:**

miR-330-3p was significantly downregulated in PPUI patients, with low expression predicting PPUI. In HUFs, miR-330-3p overexpression inhibited IL-1β-induced hyperproliferation, inflammation, and ECM degradation. Overexpression of MMP2 counteracted the influence of miR-330-3p mimic on HUFs.

**Conclusion:**

miR-330-3p is a potential biomarker for PPUI and regulates the function of urethral fibroblasts by targeting MMP2.

## Introduction

Prostate cancer, the second most diagnosed cancer in men worldwide, has seen a steady increase in survival rates due to early detection and refined surgical techniques such as robot-assisted radical prostatectomy [1]. Post-prostatectomy urinary incontinence (PPUI) is a severe and debilitating complication that significantly impairs patients’ physical condition, mental health and social activities. It is defined by involuntary urine leakage lasting more than six months post-surgery [2]. Despite progress in nerve-sparing techniques and preoperative risk stratification, up to 30% of patients report moderate-to-severe incontinence [3]. The pathophysiology of PPUI is multifactorial, involving anatomical disruption of the urethral sphincter, dysfunction of pelvic floor muscles, and neural injury sustained during prostatectomy [4]. Currently, the treatment of PPUI include conservative approaches (e.g., patient education and pelvic floor muscle exercises) and surgical interventions [5]. However, their efficacy remains limited, highlighting the urgent need to explore new therapeutic targets and mechanisms.

In recent years, research into microRNAs (miRNAs) in prostate cancer has made substantial progress [6, 7]. In addition, miRNAs regulate the expression of multiple genes and are key players in tissue repair and regeneration processes [8]. Furthermore, recent advancements in molecular pathophysiology have highlighted miRNAs as critical regulators of urinary incontinence. For instance, miR-93 has been shown to mediate the collagen expression in stress urinary incontinence [9]. Furthermore, miR-133a-3p is downregulated in patients with PPUI and exhibits high diagnostic potential for PPUI [10]. Among the numerous miRNAs, miR-330-3p has drawn considerable attention due to its potential role in cancer biology. For instance, prior studies have demonstrated that miR-330-3p acts as a promising biomarker for glioma [11]. Additionally, miR-330-3p inhibits the progression of ovarian cancer [12] and suppresses liver cancer cell migration [13]. Moreover, miR-330-3p has been implicated in regulating epithelial-to-mesenchymal transition in prostate cancer, a process critical for prostate cancer metastasis and tissue remodeling, which may be relevant to the development of PPUI [14]. Notably, miR-330-3p has been found to be downregulated in individuals with stress urinary incontinence [15]. As previously mentioned, research indicates that miR-330-3p is closely associated with both prostate cancer and urinary incontinence. However, the aberrant expression of miR-330-3p in PPUI patients and its clinical significance in this context remain to be thoroughly investigated.

Matrix metalloproteinase 2 (MMP2) is a zinc-dependent endopeptidase that has been implicated in numerous biological processes, including tissue remodeling, angiogenesis, and cancer metastasis [16–18]. Studies have shown that MMP2 is abnormally overexpressed in prostate cancer and plays a pivotal role in regulating prostate cancer metastasis [19, 20]. Furthermore, research has revealed that polymorphic genetic variations in the MMP2 gene significantly contribute to the initiation and progression of prostate cancer [21, 22]. Additionally, multiple studies have demonstrated that MMP2 mediates the pathological processes underlying stress urinary incontinence [23, 24]. In a study conducted by Hou R et al., it was reported that miR-330-3p exerts targeted regulation on MMP2 [25]. However, the interaction between miR-330-3p and MMP2 in PPUI remains to be confirmed.

Consequently, by analyzing the expression changes of miR-330-3p in patients with PPUI and combining clinical data, its diagnostic potential as a biomarker was evaluated. Meanwhile, to further elucidate the specific role of miR-330-3p in the pathogenesis of PPUI, in vitro cell experiments and molecular biology techniques were employed to investigate the effects of miR-330-3p on the proliferation of urethral fibroblasts, inflammatory responses, and ECM remodeling. Additionally, the interaction between miR-330-3p and MMP2 was validated to clarify its regulatory network in PPUI.

## Materials and methods

### Clinical specimens

The sample size was calculated using G*Power software, with the effect size d set at 0.8, α at 0.05, and power at 0.8, the calculated sample size was 128. This study enrolled 135 older adults diagnosed with prostate cancer who underwent radical prostatectomy at *Affiliated Hospital of Jiangnan University*. The cohort comprised two distinct groups: 49 patients who developed postoperative urinary incontinence and 86 patients who were without urinary incontinence (Urine control). Inclusion criteria: (1) Prostate cancer patients without distant metastasis before surgery. (2) No history of preoperative urinary incontinence. (3) Aged between 55 and 75. Exclusion criteria: (1) Patients with primary tumors at other sites; (2) Patients with mental illness. (3) Patients with other urinary system diseases. (4) Patients with neurogenic bladder or reduced bladder function.

Basic data collection and recording of all patients were carried out by the nursing staff, and 5 mL of fasting blood was collected from all patients. After all samples were centrifuged at 3000 rpm for 10 min, the serum was retained and stored at -80 degrees Celsius for future use. In addition, the nursing staff is responsible for explaining the postoperative precautions for all patients after surgery and assisting patients with rehabilitation training.

This study received approval from the Ethics Review Committee of *Affiliated Hospital of Jiangnan University*, and all participants provided informed consent forms. Furthermore, this study was conducted in accordance with the Declaration of Helsinki.

### RT-qPCR

Total RNA was extracted by using TRIzol reagent (Invitrogen, CA, USA). The purity of isolated RNA was assessed via the A260/A280 ratio. Subsequently, 1 µg of high-quality RNA was reverse transcribed into cDNA using the PrimeScript RT Reagent Kit (Applied Biosystems, CA, USA). Quantitative PCR (qPCR) was conducted using the ABI Prism 5700 Sequence Detection System (Applied Biosystems, CA, USA). GAPDH was utilized as internal controls, with data analysis conducted employing the 2^−△△Ct^ method.

### Cell culture and transfection

The 293T cells (Peking Union Cell Bank, China) and immortalized human urethral fibroblasts (HUFs) (Xinrun Biotechnology, Jiangsu, China) were maintained in DMEM medium supplemented with 10% fetal bovine serum (FBS) and 1% penicillin-streptomycin solution in 37℃, 5% CO_2_ environment.

To simulate the inflammatory environment of PPUI, the cells were treated with interleukin-1β (IL-1β) at a concentration of 10 ng/mL for 24 h [26]. Subsequently, the cells were transfected with miR-330-3p mimic, mimic NC, miR-330-3p inhibitor, inhibitor NC, MMP2 overexpression vector (oe-MMP2) or the corresponding empty vector (oe-NC) (GenePharma Co., Ltd, China) using Lipofectamine 3000 (Invitrogen, CA, USA). After 48 h of transfection, the cells were harvested for further analysis.

### Luciferase reporter assays

The wild type or mutant 3’-UTR of MMP2 was cloned into the pmiR GLO™ vector (Promega, Madison, WI, USA) to construct wild-type MMP2 (wt-MMP2) and mutant MMP2 (mut-MMP2) constructs. Subsequently, 293T cells were seeded into 24-well plates and cultured for 24 h under standard conditions. Thereafter, Lipofectamine 3000 (Invitrogen, CA, USA) was used to co-transfect the cells with miR-330-3p mimic/inhibitor or their respective negative controls (mimic NC/inhibitor NC), along with either wt-MMP2 or mut-MMP2. Cells were harvested and lysed 48 h post-transfection to determine luciferase activity.

### Cell counting Kit-8 (CCK-8) assay

Following transfection, HUFs were trypsinized, collected, and subsequently seeded into 96-well plates at a density of 2 × 10^3^ cells per well. The cell viability was assessed at multiple time points using the CCK-8 kit (Dojindo, Kumamoto, Japan). Prior to initiating of the assay, 10 µl of CCK-8 regent was added into each well, followed by incubation at 37 ℃ for 2 h. The optical density (OD) at 450 nm was measured utilizing a microplate reader (Thermo Fisher Scientific, USA).

### ELISA assay

The culture supernatant was harvested, and the concentrations of interleukin − 6 (IL-6), tumor necrosis factor-α (TNF-α), and IL-1β were quantified using ELISA with a commercially available kit (BD Biosciences, USA).

### Western blot

Total protein was extracted from HUFs using RIPA buffer (Beyotime, Shanghai, China), followed by quantification of protein concentration via BCA assay. Protein samples were separated by gel electrophoresis and subsequently transferred onto a PVDF membrane (Beyotime, Shanghai, China). The membrane was blocked with 5% skim milk for 1 h at room temperature. Following blocking, the membrane was incubated overnight at 4 °C with primary antibodies, including anti-tissue inhibitor of metalloproteinase-1 (TIMP-1) (ab211926, Abcam, USA), anti-MMP-2 (ab92536, Abcam, USA), and anti-matrix metalloproteinase 9 (MMP-9) (ab76003, Abcam, USA). After primary antibody incubation, the membrane was treated with horseradish peroxidase-conjugated secondary antibodies for 1 h at room temperature. The intensity of the bands was quantified using ImageJ software with β-actin (ab8226, Abcam, USA) as the internal control.

### Statistical analysis

Statistical analysis and data visualization were performed using GraphPad Prism 9.0 (GraphPad Software Inc., CA, USA) and SPSS 26.0 (IBM Corp., NY, USA) software. Data were expressed as mean ± standard deviation. All continuous variables were evaluated for normality using the Shapiro-Wilk test. For continuous variables, the T-test was used to analyze the statistical differences between two groups, and one-way or two-way ANOVA was used to analyze the statistical differences among multiple groups. Categorical variables were analyzed by chi-square test. The receiver operating characteristic (ROC) curve was plotted using GraphPad Prism 9.0 to evaluate the predictive effect of miR-330-3p on PPUI, and the significance of the area under the curve (AUC) was tested by the Wilson/Brown method. Multivariate logistic regression analysis was used to determine the independent risk factors for the occurrence of PPUI by using SPSS 26.0, with overall significance of the model evaluated by the likelihood ratio test, and the significance of each variable coefficient determined by the Wald test. Correlation was analyzed by Pearson correlation coefficient. In cell experiments, each group was repeated three times. Statistical significance was defined as *P* < 0.05.

## Result

### Comparison of pathological data between the two groups of patients

The pathological data of the two groups, urinary control (*n* = 86) and urinary incontinence (*n* = 49), were compared (Table 1). Significant differences were observed in age (*P* = 0.008) and body mass index (BMI) (*P* = 0.017) between the two groups. Specifically, the age and BMI of patients with urinary incontinence were greater than those of the patients without urinary continence. Additionally, the prostate volume was significantly larger in the urinary incontinence group than in the urinary control group (*P* = 0.004). Moreover, a higher proportion of patients in the urinary incontinence group had undergone transurethral prostate surgery (*P* = 0.002), while lower proportions had preserved maximum urethral length preservation (MULP) (*P* = 0.015) and maintained a complete bladder neck (*P* = 0.028). No significant differences were found in Gleason score (*P* = 0.209), preoperative prostate-specific antigen (PSA) levels (*P* = 0.561), hypertension (*P* = 0.663), and tumor staging (*P* = 0.172) between the two groups. These findings suggest that age, BMI, preoperative prostate volume, history of transurethral prostate surgery, MULP preservation, and complete bladder neck preservation may be significant factors associated with urinary incontinence following surgery.

**Table 1:** Comparison of pathological data between the two groups

Items	Urinary control (*n* = 86)	Urinary incontinence (*n* = 49)	*P* value
Age (years)			0.008
< 65	43 (50.00)	13 (26.53)	
≥ 65	43 (50.00)	36 (73.47)	
BMI			0.017
< 24 kg/m^2^	55 (63.95)	21 (42.86)	
≥ 24 kg/m^2^	31 (36.05)	28 (57.14)	
Gleason score			0.209
< 7 points	50 (58.14)	23 (46.94)	
≥ 7 points	36 (41.86)	26 (53.06)	
Preoperative prostate volume			0.004
<30 ml	38 (44.19)	9 (18.37)	
30–75 ml	34 (39.53)	33 (67.34)	
>75 ml	14 (16.28)	7 (14.29)	
Preoperative PSA			0.561
<10 ng/ml	20 (23.26)	8 (16.33)	
10–20 ng/ml	37 (43.02)	21 (42.86)	
>20 ng/ml	29 (33.72)	20 (40.81)	
Hypertension			0.663
no	61 (70.93)	33 (67.35)	
yes	25 (29.07)	16 (32.65)	
Tumor staging			0.172
< T3a phase	64 (74.42)	31 (63.27)	
≥T3a phase	22 (25.58)	18 (36.73)	
History of transurethral prostate surgery			0.002
no	63 (73.26)	23 (46.94)	
yes	23 (26.74)	26 (53.06)	
MULP keeping			0.015
no	9 (10.47)	13 (26.53)	
yes	77 (89.53)	36 (73.47)	
Complete bladder neck preserving			0.028
no	14 (16.28)	16 (32.65)	
yes	72 (83.72)	33 (67.35)	

BMI, Body Mass Index; PSA, Prostate-Specific Antigen; MULP, maximum urethral length. The data is presented as n (%), *P* < 0.05 indicates a significant difference

### Expression of miR-330-3p in patients after prostatectomy and its predictive value for PPUI

**Fig. 1:**
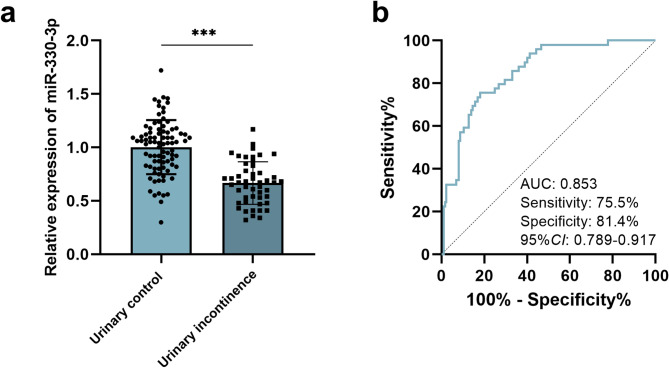
Expression of miR-330-3p in patients after prostatectomy and its predictive value for PPUI. ***p<0.001. The expression of miR-330-3p in the urinary continence group (*n* = 86) and urinary incontinence patients (*n* = 49) (**a**). The ROC curve of miR-330-3p for predicting PPUI (**b**)

Among patients who underwent prostate cancer surgery, compared with those in the urinary control group, serum miR-330-3p was decreased in patients with urinary incontinence (*P*<0.001, 95%CI = 0.42 − 0.25) (Fig. 1a). ROC analysis revealed that low expression of miR-330-3p could effectively distinguish patients with urinary continence from those with urinary control patients. The AUC of ROC curve was 0.853 (95%CI = 0.79–0.92), the sensitivity and specificity were 75.5% and 81.4%, respectively (Fig. 1b), suggesting that miR-330-3p may serve as a reliable biomarker for identifying patients of developing PPUI.

### miR-330-3p may be a risk factor for PPUI

**Fig. 2:**
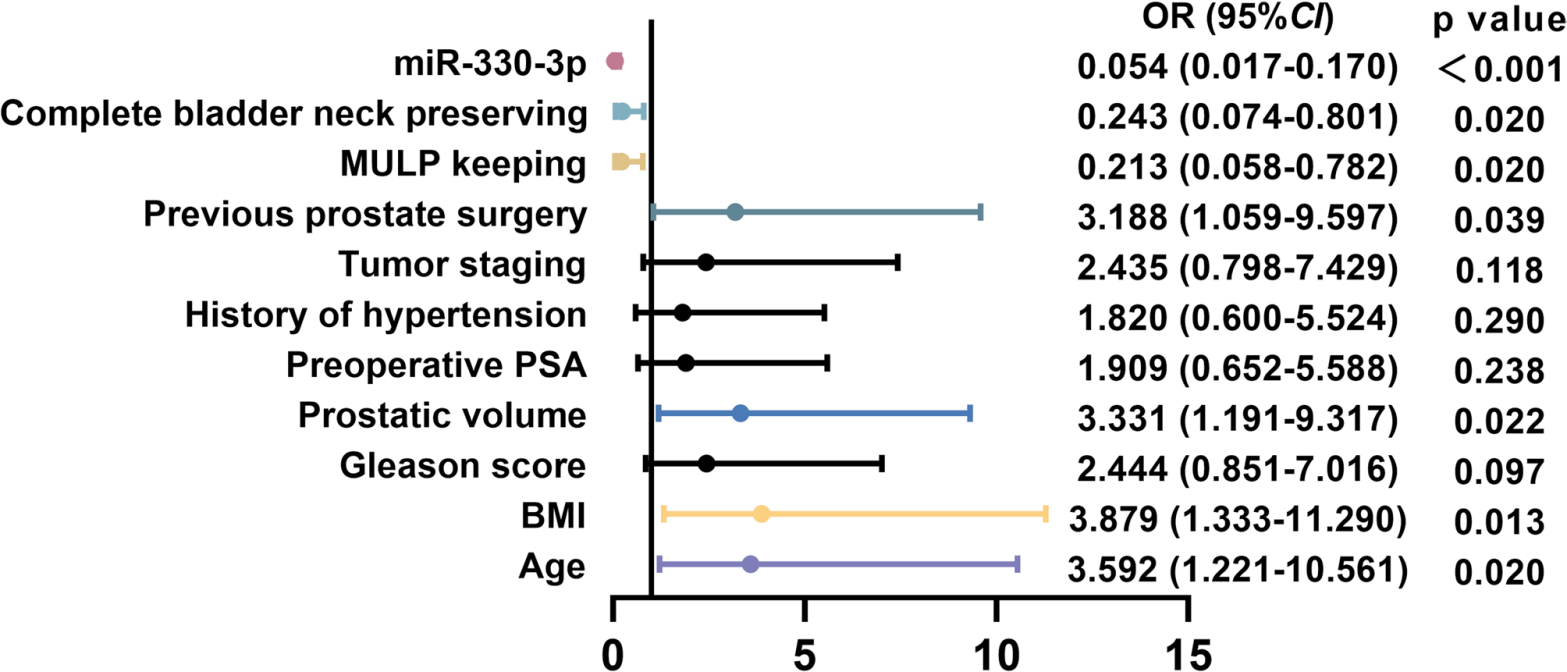
Logistic regression analysis of risk factors for postoperative urinary incontinence in patients with prostate cancer

The correlation between miR-330-3p expression and various pathological features in PPUI patients was analyzed (Table 2). PPUI patients were stratified into low-expression (*n* = 26) and high-expression (*n* = 23) groups based on the average level of miR-330-3p. Significant correlation was observed between miR-330-3p expression and age (*P* = 0.011), BMI (*P* = 0.017), preoperative prostate volume, history of transurethral prostate surgery (*P* = 0.016), MULP (*P* = 0.044) and complete bladder neck preservation (*P* = 0.032) (Table 2). These results highlighted the potential role of miR-330-3p as a biomarker associated with specific pathological features in this patient population. Furthermore, logistic regression analysis revealed that miR-330-3p might serve as a risk factor for urinary incontinence in prostate cancer patients following radical surgery (OR = 0.054, 95%CI = 0.017–0.170, *P*<0.001) (Fig. 2).

**Table 2:** Correlation between the expression of miR-330-3p and pathological features

Items	patients (*n* = 49)	miR-330-3p expression		*P* value
		low (*n* = 26)	high (*n* = 23)	
Age (years)				0.011
< 65	13	3	10	
≥ 65	36	23	13	
BMI				0.017
< 24 kg/m^2^	21	7	14	
≥ 24 kg/m^2^	28	19	9	
Gleason score				0.206
< 7 points	23	10	13	
≥ 7 points	26	16	10	
Preoperative prostate volume				0.040
<30 ml	9	2	7	
30–75 ml	33	18	15	
>75 ml	7	6	1	
Preoperative PSA				0.217
<10 ng/ml	8	2	6	
10–20 ng/ml	21	12	9	
>20 ng/ml	20	12	8	
Hypertension				0.755
no	33	17	16	
yes	16	9	7	
Tumor staging				0.146
< T3a phase	31	14	17	
≥T3a phase	18	12	6	
History of transurethral prostate surgery				0.016
no	23	8	15	
yes	26	18	8	
MULP keeping				0.044
no	13	10	3	
yes	36	16	20	
Complete bladder neck preserving				0.032
no	16	12	4	
yes	33	14	19	

BMI, Body Mass Index; PSA, Prostate-Specific Antigen; MULP, maximum urethral length. *P* < 0.05 indicates a significant difference

### MMP2 may serve as a target gene of miR-330-3p

**Fig. 3:**
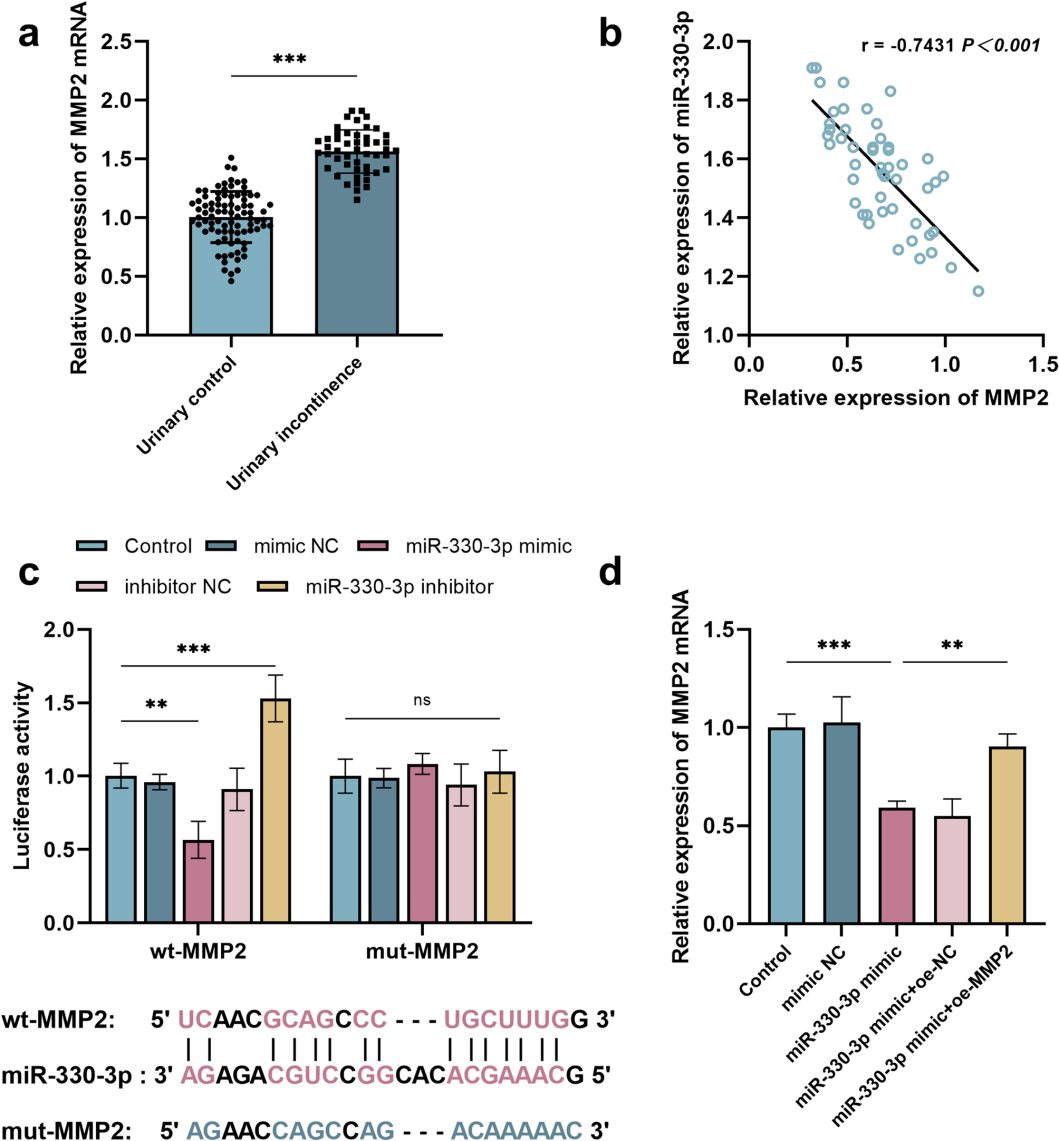
MMP2 may serve as a target gene of miR-330-3p. ***p<0.001, **p<0.01. Each group of experiments was conducted in triplicate. The expression of MMP2 in the urinary continence group (*n* = 86) and urinary incontinence patients (*n* = 49) (**a**). The correlation between the expression levels of miR-330-3p and MMP2 in patients with PPUI (**b**). The relationship between miR-330-3p and MMP2 was verified by using the dual-luciferase reporter gene assay (**c**). The relationship between miR-330-3p and MMP2 was verified by using RT-qPCR (**d**)

The expression of MMP2 was upregulated in patients with urinary incontinence (*P*<0.001, 95%CI = 0.49–0.63) (Fig. 3a), and a negative correlation existed between MMP2 and miR-330-3p levels in these patients (*r*= -0.7431, *P*<0.001, 95%CI= -0.85 to -0.58) (Fig. 3b). The binding sites between miR-330-3p and MMP2 were predicted by using starBase database, which were further validated by dual-luciferase reporter gene assay. Specifically, the miR-330-3p mimic significantly reduced the luciferase activity of wt-MMP2 (*P*<0.01, 95%CI = 0.15–0.72), whereas the miR-330-3p inhibitor increased its activity markedly (*P*<0.001, 95%CI= -0.81 to -0.24) (Fig. 3c). Furthermore, the interaction between miR-330-3p and MMP2 was also confirmed by RT-qPCR analysis. Overexpression of miR-330-3p led to a reduction in endogenous MMP2 expression levels in cells (*P*<0.001, 95%CI = 0.18–0.63), and co-transfection with an MMP2 overexpression vector bypassed this suppressive effect, leading to elevated MMP2 levels (*P*<0.01, 95%CI= -0.53 to -0.09) (Fig. 3d).

### MMP2 overexpression counteracted the effects of miR-330-3p on the proliferation, inflammation and extracellular matrix (ECM) remodeling of HUFs

**Fig. 4:**
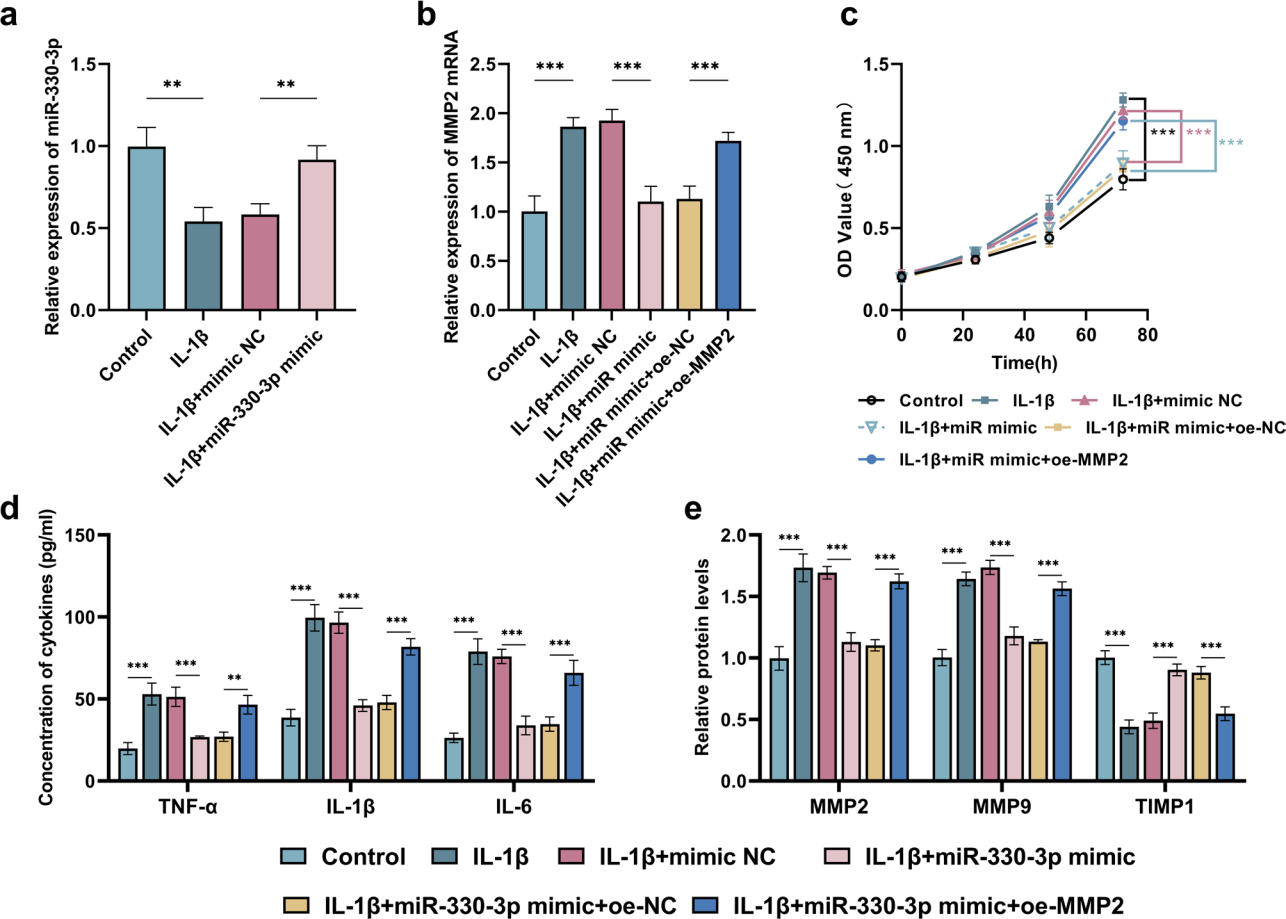
Overexpression of MMP2 counteracted the suppressive influence of miR-330-3p mimic on the proliferation, inflammation and ECM remodeling of HUFs. ***p<0.001, **p<0.01. Each group of experiments was conducted in triplicate. The expression of miR-330-3p in HUFs transfected with miR-330-3p mimic (**a**). The expression of MMP2 in HUFs transfected with miR-330-3p mimic and oe-MMP2 (**b**). Overexpression of MMP2 counteracted the effects of miR-330-3p on the proliferation (**c**), inflammation (**d**) and ECM remodeling (**e**) of HUFs

The results demonstrated that treatment of HUFs with IL-1β led to a significant reduction in miR-330-3p expression level (*P*<0.01, 95%CI = 0.22–0.69). Furthermore, transfection with miR-330-3p mimic resulted in a notable recovery of its expression level (*P*<0.01, 95%CI= -0.57 to -0.10) (Fig. 4a). Conversely, after IL-1β intervention in HUFs, the expression level of MMP2 was significantly upregulated (*P*<0.001, 95%CI= -1.20 to -0.52). Transfection with miR-330-3p mimic led to a notable decrease in levels of MMP2 (*P*<0.001, 95%CI = 0.48–1.16). Overexpression of MMP2 counteracted the suppressive influence of miR-330-3p mimic on MMP2 expression (*P*<0.001, 95%CI= -0.93 to -0.25) (Fig. 4b). After IL-1β intervention, the proliferation of HUFs was significantly enhanced (*P*<0.001, 95%CI = 0.32–0.58), miR-330-3p overexpression attenuated the IL-1β-induced increase in HUFs proliferation (*P*<0.001, 95%CI= -0.40 to -0.14) (Fig. 4c). IL-1β significantly increased the concentrations of inflammatory factors in HUFs, as evidenced by elevated levels of TNF-α (*P*<0.001, 95%CI= -46.41 to -20.00), IL-6 (*P*<0.001, 95%CI= -74.04 to -47.63), and IL-1β (*P*<0.001, 95%CI= -65.82 to -39.40). However, miR-330-3p mimic attenuated this increase in inflammatory factor concentrations (TNF-α: *P*<0.001, 95%CI = 11.28–37.69; IL-1β: *P*<0.001, 95%CI = 37.35–63.76; IL-6: *P*<0.001, 95%CI = 28.83–55.24) (Fig. 4d). After IL-1β treatment of HUFs, significant changes were observed in the protein expressions of ECM remodeling markers. Specifically, the levels of MMP2 (*P*<0.001, 95%CI= -0.89 to -0.58) and MMP9 (*P*<0.001, 95%CI= -0.79 to -0.48) were notably enhanced, while the protein expression of TIMP1 was decreased (*P*<0.001, 95%CI = 0.40–0.72). Transfection of miR-330-3p mimic led to a reduction in the protein levels of both MMP2 (*P*<0.001, 95%CI = 0.41–0.72) and MMP9 (*P*<0.001, 95%CI = 0.40–0.71) and an increase in TIMP1 protein levels (*P*<0.001, 95%CI= -0.56 to -0.26) (Fig. 4e). However, Overexpression of MMP2 counteracted the suppressive influence of miR-330-3p mimic on the beneficial effects on cell proliferation (*P*<0.001, 95%CI = 0.16–0.42), the secretion of inflammatory factors (TNF-α: *P*<0.01, 95%CI= -32.73 to -6.31; IL-1β: *P*<0.001, 95%CI= -47.12 to -20.71; IL-6: *P*<0.001, 95%CI= -44.43 to -18.01) and ECM remodeling (MMP2: *P*<0.001, 95%CI= -0.67 to -0.36; MMP9: *P*<0.001, 95%CI= -0.59 to -0.27; IL-6: *P*<0.001, 95%CI = 0.18–0.49) (Fig. 4c and e).

## Discussion

An increasing body of evidence has shown that miRNAs play critical roles in postoperative complications associated with cancer. For instance, miR-210 has been identified as a significant risk factor for postoperative delirium in patients undergoing radical gastrectomy for gastric cancer [27]. Additionally, miR-1-3p exhibits differential expression in patients experiencing anthracycline-induced liver injury following breast cancer surgery [28]. This study for the first time revealed the clinical value of miR-330-3p in PPUI and its potential molecular mechanism.

The levels of miR-330-3p were significantly reduced in patients with PPUI, and its low expression level may serve as a predictive tool for differentiating urinary incontinence patients from those urinary control patients. This finding suggests that miR-330-3p may potentially function as a predictive tool for postoperative urinary incontinence and provide novel targets for early intervention. Notably, the expression level of miR-330-3p is significantly correlated with multiple clinical characteristics (such as age, BMI, preoperative prostate volume, history of transurethral prostate surgery, MULP preservation and complete bladder neck preservation). This further supports its clinical relevance as a risk factor for postoperative urinary incontinence. In particular, the preservation status of MULP and the bladder neck is closely related to the recovery of urinary control function [29, 30], and miR-330-3p may affect this process by regulating the repair ability of urethral supporting tissues.

Studies have shown that members of the miR-330 family play critical roles in ECM remodeling, inflammatory responses and cell proliferation. For instance, miR-330-5p has been shown to alleviate intervertebral disc degeneration by regulating ECM remodeling [31]. Additionally, miR-330-5p suppresses NLRP3 inflammasome formation during myocardial ischemia-reperfusion injury [32]. Furthermore, it has been established that miR-330-3p directly targets TNF-α and inhibits the production of matrix-degrading enzymes [33]. In our study, the low expression of miR-330-3p may exacerbate fibrosis and abnormal repair in urethral tissues through similar mechanisms, potentially leading to urinary incontinence.

Further mechanistic studies demonstrated that miR-330-3p regulates the function of urethral fibroblasts by directly targeting MMP2, which also validates the previous research results [25]. MMP2 serves as a key enzyme in ECM degradation, the overexpression of MMP2 can lead to tissue fibrosis and abnormal structural remodeling [34]. The fundamental constituents of the urethral support tissue are primarily composed of connective tissues that encompass a substantial quantity of ECM alongside a minimal presence of cellular elements. The predominant element within the ECM is collagen, which encompasses both collagen I and collagen III [35, 36]. Abnormal deposition of ECM is often accompanied by increased expression levels of MMP2 and MMP9, although their activities may be regulated by inhibitory factors such as TIMPs This regulatory mechanism may lead to excessive ECM degradation, thereby affecting tissues repair and remodeling process [34]. This study revealed that in the inflammatory microenvironment stimulated by IL-1β, HUFs fibroblasts exhibited excessive proliferation. Additionally, the expression of MMP2 and MMP9 increased to cope with the abnormal deposition of ECM. Moreover, the expression of TIMP1 decreased, indicating enhanced ECM degradation activity, which might lead to the destruction of tissue structure and abnormal function. More importantly, overexpression of miR-330-3p improved the excessive proliferation of HUFs and alleviated the cell inflammation induced by IL-β. Additionally, Overexpression of MMP2 counteracted the suppressive influence of miR-330-3p mimic on the secretion of inflammatory factors and the beneficial effects on cell proliferation and ECM remodeling. This indicates that MMP2 may interfere with the regulatory effect of miR-330-3p.

Undoubtedly, nursing interventions play an indispensable role in the recovery from PPUI. Studies in this field have demonstrated that measuring quality of life is as critical as the treatment itself when providing comprehensive care [37]. Furthermore, pelvic floor muscle training exerts a positive influence on the recovery from PPUI [38]. Moreover, psychological nursing intervention improved the anxiety and depression symptoms of patients with severe urinary incontinence after radical prostatectomy and enhanced their quality of life [39]. In this study, the nursing staff provided active psychological counseling and targeted rehabilitation training to patients, which contributed significantly to preventing urinary incontinence and facilitating its recovery.

The current evidence indicates that miRNAs affect the progression of diseases through multiple secretion pathways. For example, the miR-500a-3p secreted by cancer-associated fibroblast exosomes promotes prostate cancer metastasis [40]. The miRNAs secreted by the extracellular vesicles of cells derived from the tissue sources related to prostate cancer and biological fluids may become non-invasive diagnostic indicators for prostate cancer [41]. Our in vitro experimental data showed that miR-330-3p expression is decreased in HUFs stimulated by IL-1β, suggesting that miR-330-3p in serum may partially originate from dysfunctional urethral tissue. Additionally, this study found that miR-330-3p also regulates the inflammatory/remodeling process, indicating that miR-330-3p may be involved in the systemic response after prostatectomy. Of course, more data is needed to prove this. Future experiments should directly compare the levels of miR-330-3p in matched serum, urine exosomes, and urethral biopsy samples to clarify its source.

Based on the findings of this study, multiple possibilities are provided for the treatment of PPUI. For instance, the combination of serum miR-330-3p and existing urodynamic indicators may optimize the classification of PPUI subtypes (such as fibrosis-dominant type vs. nerve injury type). Local delivery of miR-330-3p mimics to the urethral sphincter via nanocarriers (such as liposomes) may restore the balance of MMP2 and be used for anti-fibrotic treatment. Additionally, dynamic monitoring of miR-330-3p levels may evaluate the efficacy of clinical treatment and enable individualized adjustments.

In conclusion, the expression level of miR-330-3p was significantly decreased in PPUI patients. This suggested that miR-330-3p may play an important role in the pathogenesis of PPUI. The low expression level of miR-330-3p was correlated with the occurrence of PPUI, indicating that miR-330-3p may become an important biomarker for predicting the risk of PPUI. Overexpression of miR-330-3p significantly reduced excessive cell proliferation, which is usually closely related to the inflammatory response. miR-330-3p also reduced the release of inflammatory factors, thereby reducing the severity of the inflammatory response. Moreover, overexpression of miR-330-3p inhibited the excessive degradation of ECM, which is considered to be one of the key factors leading to tissue structure destruction and functional impairment. Overexpression of MMP2 counteracted the protective effect brought by miR-330-3p. This discovery not only revealed the interaction relationship between miR-330-3p and MMP2, but also provided a theoretical basis for further developing treatment strategies for PPUI. Clinically, this highlights miR-330-3p as a potential therapeutic target—strategies to restore miR-330-3p expression or inhibit MMP2 activity may offer new approaches for PPUI prevention or treatment.

Undeniably, this study also has certain limitations. Firstly, the current study mainly focused on in vitro experiments and the molecular mechanism level, and the understanding of the complex pathophysiological process of PPUI is still not comprehensive and in-depth. Due to the lack of in vivo validation, the specificity and dynamic change pattern of serum miR-330-3p as a biomarker remain unclear, which may affect the accuracy of its clinical application. Future research needs to conduct in vivo models (such as PPUI animal models) to verify the impact of miR-330-3p/MMP2 axis on the structure and function of urethral tissue. Additionally, the current study mainly focused on the changes in ECM components, but systematic exploration of other possible key mechanisms, such as nerve function damage, inflammatory response, and immune regulation, has not yet been conducted. This may lead to an incomplete understanding of the pathogenesis of PPUI. Therefore, the future study plans to further expand the research scope, not only deeply analyzing the expression changes of ECM-related components under different conditions, but also comprehensively evaluating the role mechanisms of nerve function, inflammatory factors, and other potential signaling pathways in PPUI. Furthermore, as this study did not directly compare the levels of miR-330-3p in urethral tissue and serum, which may lead to an inability to distinguish whether miR-330-3p levels is due to urethral injury or a systemic response. Future research will verify the relationship between local and systemic miR-330-3p through animal models or clinical paired samples (such as comparing urethral tissue and serum obtained during surgery), thereby enhancing the clinical translation value of this study.

## Data Availability

The datasets generated during and/or analysed during the current study are available from the corresponding author on reasonable request.

## Abbreviations

PPUI: Post prostatectomy urinary incontinence
MMP2: Matrix metalloproteinase 2
RT-qPCR: Reverse transcription PCR
ROC: Receiver operating characteristic
HUFs: Human urethral fibroblasts
CCK-8: Cell counting kit-8
ELISA: Enzyme-linked immunosorbent assay
ECM: Extracellular matrix
FBS: Fetal bovine serum
DMEM: Dulbecco’s modified eagle’s medium
IL-1β: Interleukin-1β
IL-6: Interleukin-6
TNF-α: Tumor necrosis factor-α
TIMP-1: Metalloproteinase-1
MMP-9: Matrix metalloproteinase 9
MULP: Maximum urethral length
PSA: Prostate-specific antigen
BMI: Body mass index
